# Supplementary material for: Patterns of Intron Gain and Loss in Fungi
Source: PLoS Biol. 2004 Nov 30;2(12):e422. doi: 10.1371/journal.pbio.0020422 (PMC532390; doi:10.1371/journal.pbio.0020422)
Supplement: Table S1 — Also available at http://genes.mit.edu/NielsenEtAl/. (4.3 MB ZIP). [file pbio.0020422.st001.zip › NielsenEtAl/html/304.html]

AN8063.1.NCU07959.1.MG05139.1.FG00406.1


```
 CLUSTAL W (1.82) Multiple Sequence Alignments - Introns Inserted


Sequence 1: MG05139.1	476 aa
Sequence 2: FG00406.1	475 aa
Sequence 3: NCU07959.1	484 aa
Sequence 4: AN8063.1	473 aa
Alignment Length: 498 aa
Number Identitical Residues: 138 aa
Alignment Score (without introns) 7845


MG05139.1 	MQLNTQLSALLGLL-LPTLASAETVLGVFVFHRHGDRTAKSTPPTKLTALGAEQVHSSGE
NCU07959.1	--MHTKA---LLLASIPAATAAETILGAYIFSRHGDRTTKAYSPTKLTALGAEQVYLSGS
FG00406.1 	--MHAKLSSLLFLSSIPSFTLAEDVLGLYIFHRHGDRTAKAWKPVNFTALGADEVHSSGS
AN8063.1  	---MRRSLQLIALSAIPLQVSAEKVLGAYIFARHGDRTAKILKNTQLTDLGYNQVYLTGS
          	     :    : * ::*  . ** :** ::* ******:*    .::* ** ::*: :*.

MG05139.1 	YYRSLYVASNATSRIAGISSDVAVNSQLNVVSPIDVVLQNSGQAFLQGLYPPT-TADVQV
NCU07959.1	WYRSRYIDEKSSTRINGISSDLADLTELAVTAPADNVLQGSAGFFLQGLYPPAGSVNSQK
FG00406.1 	WYRDTYVSKDAPRKISGLSSESVALSQLDVTSPVDAVLQNSALVFLQGLYPP--TQQAET
AN8063.1  	AYHDRYINSTSSKYIDGISEDVVNLSQLTASAPADAVLQNSATGFLQGVYPPVGSVSSQE
          	 *:. *: . :.  * *:*.: .  ::* . :* * ***.*.  ****:*** .: . : 

MG05139.1 	LADGTTVQSTLGGYQYIPVNSLTTAATPSG-AEDDAWLQSGSGCAKAVASSNSYFASRDY
NCU07959.1	LANGTTIEAPLSGYQYIPVNSATVTSRGSNQAESSAWLQGNSGCPAAVNSSQAYYQSKEY
FG00406.1 	LANGSKVEAPLSGYQYIPIASVESAASDKH-SESNAWLQGNSGCTNAEASSNAYLSSSAF
AN8063.1  	LANGTTVESPLNGYQLIPLSLIDSGAS----SEDSTWLQGTTDCNRAKVSSDSYYESELY
          	**:*:.:::.*.*** **:      :     :*..:***. :.*  *  **::*  *  :

MG05139.1 	LDALESTHGFYQGLMPAINRTFKADAANFKNGYAI1YDLINVAQIHNASYDGRNLMTDPV
NCU07959.1	FELLGSTKEFYQSLLPVYESTFPSESATFKNAYSI1FDYIHVSTIHNTSIPSSNLLTPSN
FG00406.1 	DKTYKETEDFYQSLLPVIQKTYGKDEANFRNAYTI1FDLINVARIHNSSIPSDNLLKEST
AN8063.1  	KTLFEETKDFYQSLSPLINTSFSDADMSFRNAYSI~YDYLNVGSIHNTS----NTPTESQ
          	     .*. ***.* *  : ::     .*:*.*:* :* ::*. ***:*    *  . . 

MG05139.1 	LHQLSTRANQLEWNLAFNASEPVRAIAGATLAGQALRSLNATLQKPASSPRIDVQFGAYA
NCU07959.1	LQTLSDLANIHEFGLGYNASSPIRAVAGSVLASHIVSTLNSSISSS-SSQKLNIQFGPYG
FG00406.1 	LDKLYNLASIHEWNLAYNSSEPVRAIAGSVLGGQILEALEP-LAEGKKAPKVNIQFGAYG
AN8063.1  	LHQAFLLANIEQYNLAYNSSETVRAIAGSTLAAEMLMGLNETVTSQ-GEVKLHLEFGSYG
          	*.     *.  ::.*.:*:*..:**:**:.*... :  *: :: .     ::.::**.*.

MG05139.1 	TFSSFFGLAGLHEVSTDFTGIVDYASSMAFELVTNVSVPAGAVSPDDVSVRFRFVNGSAS
NCU07959.1	TFGSFFGLSQLPAVSSDFTAIVDYASSMTFELFTTSSSSS--PSENEVFVRFLFANGTAS
FG00406.1 	AFMAFFGLAGLDKASSDFMGIVDYASSMAFELVTNVTNPT----VNDVSVRFYFANGTAG
AN8063.1  	TFLSYFGLANLPAVDAAFTGIPDYASSMVWELVTNSTSDS-FPSTDEISVRFAFHNGTMT
          	:* ::***: *  ..: * .* ******.:**.*. :  :   : ::: *** * **:  

MG05139.1 	-AVRPLRAYPLFGPGESLMPWSAFQRNMAKIAVMD-TARWCELCGPASAGCAAGAAG---
NCU07959.1	-DSNPAKVYPLFGQDKTELPWTTFVSEMNKIALEGGSKEWCNACGEKTGQCADYYPA---
FG00406.1 	-ENTP-KLFPLFGDDATTLSWKDFKTGMTGFAIED-TKHWCKLCGNTDGTCASSSSGDDD
AN8063.1  	GSSSTPTTYPLYGQSSSTIPWSTFVEQTEKIAVMT-TQQWCDVCGNTDGQCASSSDTSDS
          	.   .   :**:* . : :.*. *      :*:   : .**. **   . **     ...

MG05139.1 	-TAGAAGAASGGSDGGITTPVAGVIGALVTLGLVLITGGLVMGVGGLRLVKKRDLVP---
NCU07959.1	--ESAGVAAAAKSGNGMSNAVAGVIGAMVTLGVVLGLQALILGVGGLRLTKRRKQDPSEV
FG00406.1 	DDSASSSSNSGGSSGGISKPVAGVIGALVTLVVILGVQAAVILIGGLRLVKKSTLAP---
AN8063.1  	-GNASTNSFAGSNGGGISKGVAGVIGAMVTLAVILGLEALFLLLGPFRLSKKGRLAG---
          	   .:  : :. ...*::. *******:*** ::*   . .: :* :** *:        

MG05139.1 	-------APASDKMSA---
NCU07959.1	FQVPAGKTSFSDTSSTQKA
FG00406.1 	-------AAAASAPAMAKA
AN8063.1  	-------IDRGNVVDENKD
          	          ..     .
```
